# Macroscopic CNT fibres inducing non-epitaxial nucleation and orientation of semicrystalline polymers

**DOI:** 10.1038/srep16729

**Published:** 2015-11-18

**Authors:** Hangbo Yue, Alfonso Monreal-Bernal, Juan P. Fernández-Blázquez, Javier Llorca, Juan J. Vilatela

**Affiliations:** 1 IMDEA Materials Institute, Eric Kandel 2, Getafe, Madrid 28906, Spain; 2 Department of Materials Science, Polytechnic University of Madrid, 28040 Madrid, Spain

## Abstract

In the presence of macroscopic fibres of carbon nanotubes (CNT), various semicrystalline polymers are shown to present accelerated crystallisation through the formation of a transcrystalline (TC) layer perpendicular to the fibre axis. From differential scanning calorimetry, polarized optical microscopy and X-ray diffraction we establish this to be due to much faster nucleation rates at the fibre surface. The formation of a TC layers is demonstrated for polyvinyldene fluoride, isotactic polypropylene and poly(lactic acid) in spite of the large differences in their chemistry and structure unit cells, suggesting that epitaxy in terms of lattice type or size matching is not a prerequisite. For the three polymers as well as poly(ether ether ketone), the TC layer is identically oriented with the chain axis in the lamella parallel to the CNTs, as observed by wide and small angle X-ray scattering. These results point to polymer chain orientation at the point of adsorption and the formation of a mesomorphic layer as possible steps in the fast nucleation of oriented lamella, with wetting of the CNT fibre surface by the molten semi-crystalline polymer a key condition for heterogeneous nucleation to take place.

The heterogeneous nucleation of semicrystalline polymers on the surface of a reinforcement element in a composite changes dramatically the interfacial and thus bulk properties of the composite system. In traditional high-performance fibres, the fibre surface presents a high density of nuclei which force unidirectional crystal growth perpendicular to the fibre axis, forming a region called transcrystalline (TC) layer. Some questions remain as to the origin of enhanced nucleation at the fibre surface^1^, but it is well established that the TC significantly increases fibre-matrix adhesion^2^ and results in an improvement in composite mechanical properties^3^. Macroscopic fibres made up of preferentially aligned carbon nanotubes (CNT) have also been shown to induce polymer transcrystallinity when embedded in isotactic polypropylene (PP), leading to a similar effect on composite mechanical properties^4^,^5^ and often to the preferential nucleation of the γ-phase^6^. Similarly, nanoparticles used as fillers dispersed in semicrystalline polymer matrices have been widely observed to act as nucleating agents on account of their large surface-to-volume ratio. Melt-spun CNT/PP composites, for example, were shown to crystallize faster than pure PP samples at the same temperature^7^. A similar behavior is also observed in polymer composites with graphene^8^ and other nanofillers^9^. The ability of nanocarbons (i.e. CNTs, graphene) and graphitic materials to act as nucleating agents has been attributed to various factors including: epitaxy (i.e. some degree of crystallographic registry or matching of unit cell parameters), for example when the polymer in helical crystal conformation matches low energy adsorption sites of large graphitic surfaces^10^; orientation of the polymer chains and graphitic planes without crystallographic registry, termed “soft epitaxy” and observed predominantly in CNT/polymer composites prepared from solution^11^; and the high thermal conductivity in the graphite basal plane (tube axis) producing a local temperature gradient by dissipation of excess heat from exothermal crystallisation^12^. The recent review by Laird *et al*. addresses in detail current understanding of crystallisation in CNT-polymer systems^13^. It also highlights open questions in this area, such as the role of CNT diameter, surface chemistry and epitaxy in the formation of the TC layer.

Here we present results on accelerated crystallisation of polymers with different chemistry—PP, polyvinyldene fluoride (PVDF) and poly(lactic acid) (PLA)—in the presence of macroscopic fibres made up of aligned CNTs. These systems are ideal to study CNT-polymer interactions due to the wide variety of polymer chemistry, unit cells, and surface energy (see Supplementary Table S1 online). Moreover, the fibres have a high surface area (>250 m^2^/g) and can be used to produce composites with high volume fraction (>15%) of aligned CNTs, thus enabling bulk measurements by differential scanning calorimetry (DSC), polarized optical microscopy (POM) and wide and small angle X-ray scattering (WAXS/SAXS) that provide additional information about the heterogeneous nucleation of semi-crystalline polymer, otherwise inaccessible when working with CNTs dispersed in polymer matrices. From these measurements we observe accelerated crystallisation and chain axis parallel to the CNTs (i.e. to the fibre axis) irrespective of polymer type, suggesting that epitaxy is not a prerequisite for the nucleation of the TC layer. In addition to contributing to the discussion on polymer crystal nucleation on CNTs and other nanocarbons, this work intends to set the ground for development of large polymer composite structures based on fibres of CNTs, currently produced on a kilometers/day scale in the laboratory^14^ and larger quantities in semi-industrial environments.

## Results and Discussion

### Evidence of TC layer formation for different polymers

**Figure 1:**
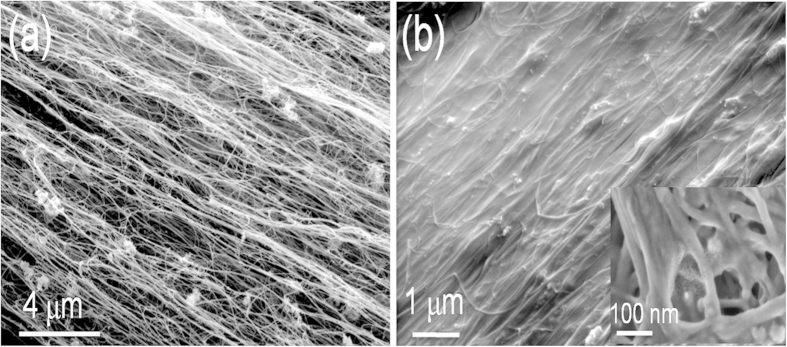
SEM micrographs of (**a**) as-spun CNT fibre and (**b**) PLA-CNT fibre composite showing coating of the CNTs by the polymer as evidence of their affinity.

The CNT fibres have a hierarchical yarn-like structure^15^ with a large specific surface (>250 m^2^/g) and porosity arising from imperfect packing of CNT bundles. Such structure provides a large surface area for interaction with liquids^16^ and polymers^17^. Fig. 1 presents SEM micrographs of the neat fibre and a composite sample (PLA/CNT fibre) showing coating of the CNT bundles by the thermoplastic as evidence of the interaction between polymer and graphitic material. This is further observed in the high-resolution electron micrograph in Fig. 1b (insert). PVDF and PP also used in this study, as well as other thermoplastics reported (e.g. PS^18^, Nylon-619, PVA20) show similar affinity with CNT fibres.

**Figure 2:**
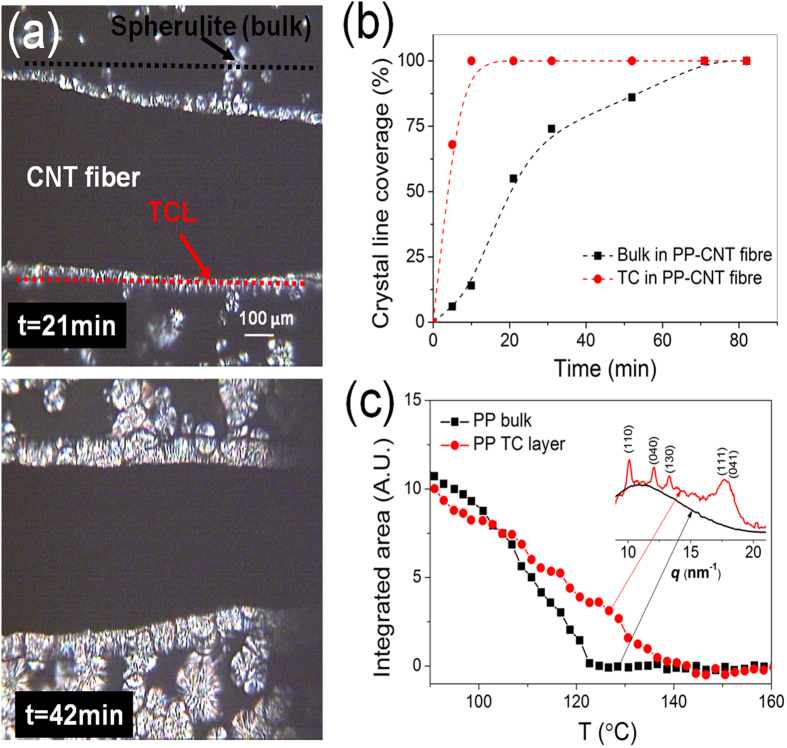
Evidence of the TC layer forming due to accelerated nucleation compared to the bulk. (**a**) Sequential micrographs of CNT fibre-PP composite during isothermal crystallisation showing the formation of a TC layer before substantial spherulite growth. (**b**) Comparison of crystal coverage over time of the fibre surface and an arbitrary line in the bulk. (**c**) Temperature dependence of crystallisation of TC layer in a CNT fibre/PP composite compared to bulk crystallisation in pure PP, obtained from the area of the PP (040) reflection in XRD diffractograms. Inset: examples of diffractograms at 138 °C for both samples.

The large surface area of the fibre provides multiple sites for heterogeneous nucleation of the semicrystalline polymers and the formation of a TC layer normal to the CNTs and thus to the fibre surface. The example in Fig. 2a presents two sequential POM micrographs during the early stages of isothermal crystallisation of PP, showing complete coverage of the fibre surface by a TC layer while the bulk of the composite is still predominantly amorphous with only a few spherulites detectable. We make a qualitative comparison of the TC and bulk crystallisation processes by plotting the fraction of linear coverage by crystal over time for the fibre edge and for an arbitrary line in the bulk (red line). Figure 2b confirms the appearance of the TC layer before substantial growth of the spherulites in the bulk. Such behavior is also observed for the other two polymers studied (PLA and PVDF).

Similarly, polymer crystallisation during cooling followed by *in-situ* XRD starts at higher temperatures in the composite sample compared to neat polymer, indicating faster crystal nucleation at the fibre surface. This can be seen in Fig. 2c, corresponding to the increase in peak intensity of the PP (040) reflection during cooling of the two samples, and which shows the TC formation some 20 °C before bulk crystallisation.

**Figure 3:**
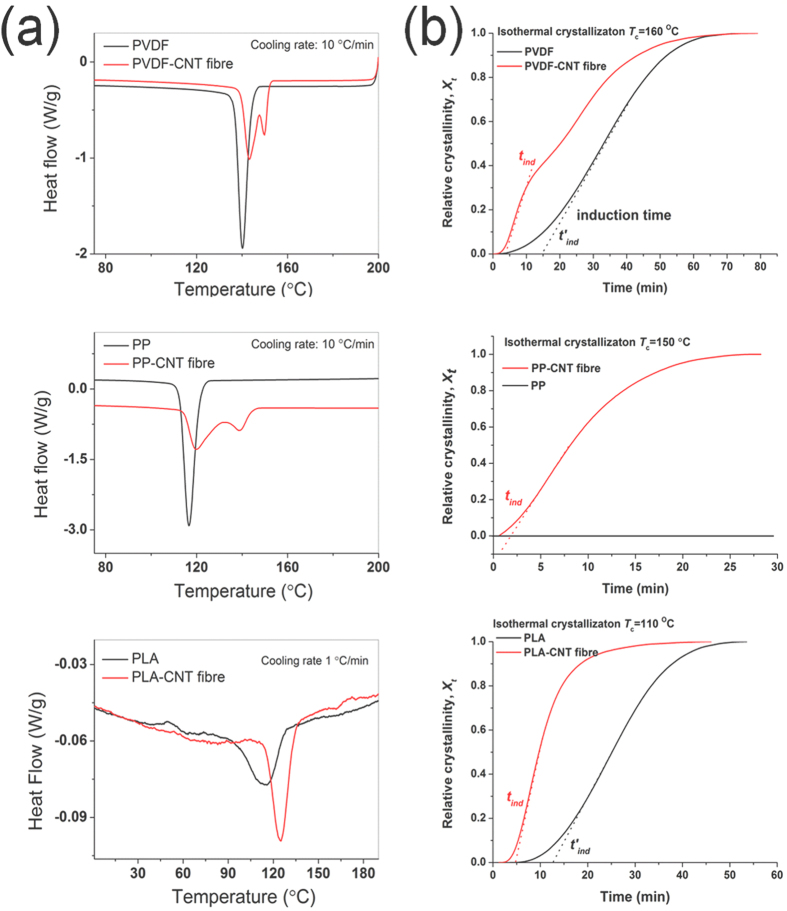
Comparison of crystallisation of pure polymers (PVDF, PP PLA) and the corresponding CNT fibre composites. (**a**) DSC curves during slow cooling from the melt, showing two crystallisation modes in the composite samples, corresponding to TC layer (higher temperature) and bulk (lower temperature). (**b**) Plots of relative crystallinity *X*_*t*_ vs time during isothermal crystallisation showing accelerated crystallisation in composites by the presence of the TC component. The onset of TC growth is taken as the induction time for nucleation *t*_*ind*_, which through its temperature dependence can be used to compare nucleation rate constants.

The large volume fraction of CNTs in this type of composites enables the nucleation of the different thermoplastics in the presence of the CNT fibre to be followed directly by DSC. Figure 3a present DSC heat flow plots during slow cooling from the melt of both pure polymers (PVDF, PP, PLA) and composites (mass fraction *m*_*f*_ = 10, 15, 13%, respectively). The TC is evidenced as an exothermic peak 10 °C to 30 °C above the temperature of bulk crystallisation. PP and PVDF present a sharper separation of crystallisation components than PLA due to the specific crystallisation kinetics of each polymer system. From similar measurements during isothermal (160 °C, 150 °C, 110 °C, respectively) crystallisation in supercooling state it is possible to extract a relative crystallinity (*X*_*t*_) at time *t* after normalizing by the crystallinity once the heat flow curve has leveled off, and to obtain a characteristic time related to the kinetics of the process. Examples are presented in Supplementary Fig. S2 online. The curves of *X*_*t*_ vs *t*, presented in Fig. 3b, have a sigmoidal shape typical of thermoplastic crystallisation, with the three composites showing accelerated crystallisation (Note that in the case of PP the growth in the presence of CNTs starts at much higher temperatures than the pure polymer). Taking the PVDF composite as an example, two crystal growth peaks occurring at different times can be clearly detected, which upon comparison with pure PVDF can be assigned to TC and bulk crystallisations at the lower and higher temperature, respectively. Similar to the time at half relative crystallisation, *t*_*1/2*_, often extracted from these plots, here we extract an induction time corresponding to the onset of crystallisation, *t*_*ind*_ (see Supplementary Fig. S3 online).

Considering the POM and XRD results above, it is clear that the two peaks in DSC can then be assigned to TC and bulk crystallisation, respectively, where the higher slope of the TC curves would evidently be indicative of faster crystallisation kinetics. The DSC data can also be used to compare the nucleation rates of the two processes. From the Avrami plots (Fig. 3b), extrapolating to *X*_*t*_ = 0 we obtain an induction time, *t*_*ind*_, which we assume to correspond to the induction time for nucleation^21^. This is equivalent to assuming that the onset for nucleation and the onset for growth detected by DSC are the same, which is a reasonable assumption for the purposes of comparing nucleation rates for the TC and bulk because the interest is in the temperature dependence of the induction time rather than in the absolute values of *t*_*ind*_.

Since the induction time is inversely proportional to the nucleation rate (*I*), one can extract the temperature dependence of *I* from that of *t*_*ind*_. This dependence should be of the form


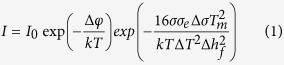


where *I*_*0*_ is a constant nucleation rate, Δφ an activation energy *k* is Boltzmann’s constant, *T* the crystallisation temperature, Δ*h*_*f*_ the heat of fusion per unit volume of crystal, *T*_*m*_ the melting point Δ*T*, the undercooling degree, and σ,σ_*e*_, Δσ the side-surface energy, fold surface energy and interfacial free energy difference function of the nucleus, respectively.

**Figure 4:**
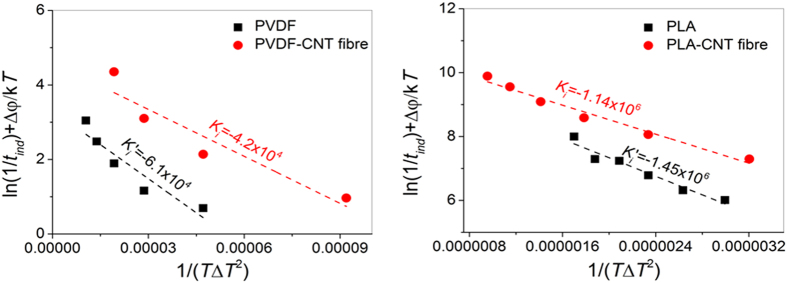
The temperature dependence of reciprocal of induction time for the calculation of nucleation kinetic constants from the slope of *ln(1/ t*_*ind*_*) + Δφ/kT vs 1/(TΔT*^*2*^). The values indicate faster nucleation of the TC than the bulk for all composites compared to pure polymer.

From a series of isothermal crystallisation experiments at different temperatures, by plotting ln*(1/t*_*ind*_) + Δφ*/kT* versus *1/(T*Δ*T*^*2*^) we obtain a slope *K*_*i*_ = *16*σσ_*e*_Δσ *T*_*m*_^2^*/k*Δ*h*_*f*_^2^, which is the factor modulating nucleation rate under isothermal conditions^21^. A similar procedure can be applied to the pure polymer data with the induction time now associated to heterogeneous nucleation in the bulk, *t*_*ind*_′ (Fig. 3b). Figure 4 presents these plots for the composite and pure polymer systems of PVDF and PLA. The linear fitting is good for PLA and more approximate for PVDF, but both show equivalent trends. The lower values of |*K*_*i*_| for the composites compared to the pure polymer confirm that accelerated crystallisation in the presence of the fibre is largely due to a faster nucleation rate at its surface, which then gives as a result the formation of the TC. Samples of PP are in line with this trend (see Supplementary Fig. S4 online), but the TC formation is too fast to extract precise values of *K*_*i*_ from isothermal experiments at different temperatures.

The dominance of either TC or bulk crystallisation is usually quantified as *A = * Δσ′*/*Δσ, taking values <1 for predominantly bulk crystallisation and >1 for predominantly TC. Assuming that the fold and side-surface energies are the same in both growth modes, *A* is equal to the ratio of the nucleation rates constants *K*_*i*_ which comes out as 1.79 and 1.69 for PVDF and PLA, respectively. Further analysis to extract values of σ, σ_*e*_ and Δσ would require knowledge of the growth rates, which although reported to be similar in the two crystallisation modes for some polymers^22^,^23^,^24^, could differ due to the specific crystal growing habits at crystal growth front, and which can be more accurately determined by continuous cooling DSC^25^.

### Nucleation and wetting

It is interesting that in spite of their differences in chemical nature the three polymers used in this work show TC nucleation behavior when in contact with the CNT fibre. Typically a simple kinetic model of crystallisation via heterogeneous nucleation includes several steps: attachment of polymer on the external surface, diffusive transport across the surface, structural transformations, crystallographic registry and nucleation^26^. However, the first question is the ability of the polymers chosen to wet the CNT fibre. Evidently that is the case for all the polymers in this work, which when in molten state or solution readily wet the porous fibre structure (see Supplementary Fig. S5 online) and can wick depending on porosity and the viscosity at the point of infiltration^27^. In fact, in view of the wide range of polymers that seem to wet CNT fibres there might be many more candidates to form a TC layer, particularly because epitaxy appears not to be a prerequisite in these systems, as shown later.

To provide a first prediction of which semicrystalline polymers could form a TC at the CNT fibre surface we compare the differences in surface free energies of the CNT-polymer interface and the pure CNT surface, which must be <0 for wetting to take place. This condition is normally approximated by partition of the surface energies into components, for example dispersive (γ^*d*^) and polar (γ^*p*^) using the Owens approximation^28^ (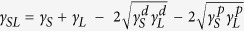), reducing the condition for wetting to


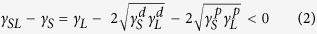


which can be calculated from literature data for the surface energy components of the polymers and CNTs. Although lacking thermodynamic rigor and ignoring entropy changes, such type of expressions accurately describe a wide range of experimental data on adhesion phenomena^29^. Table 1 presents values of γ_*SL*_ – γ_*S*_, where we have included two values of γ_*S*_ to cover a range of graphite chemistries, one for thin MWNTs with dispersive and polar components^30^ and another purely dispersive for graphite^31^. In both cases we find γ_*SL*_ – γ_*S*_ < 0, indicating a favorable interaction for the three polymers tested, in agreement with the observation of polymer melt infiltration into the fibres.

We note that while polymer miscibility is often dealt with in terms of Hildebrand or Hansen solubility parameters, these are less appropriate for CNTs because of the dependence of CNT cohesive energy density on diameter^32^. Nevertheless, the values of polymer-CNT distance in Hansen space (*R*_PLA_ = 1.6, *R*_PP_ = 8.5, *R*_PVDF_ = 7.1 MPa^1/2^) still come out in the “wetting” range (1 < *R* < 10)^32^ (see Supplementary Table S2 online).

### CNT-polymer epitaxy

**Figure 5:**
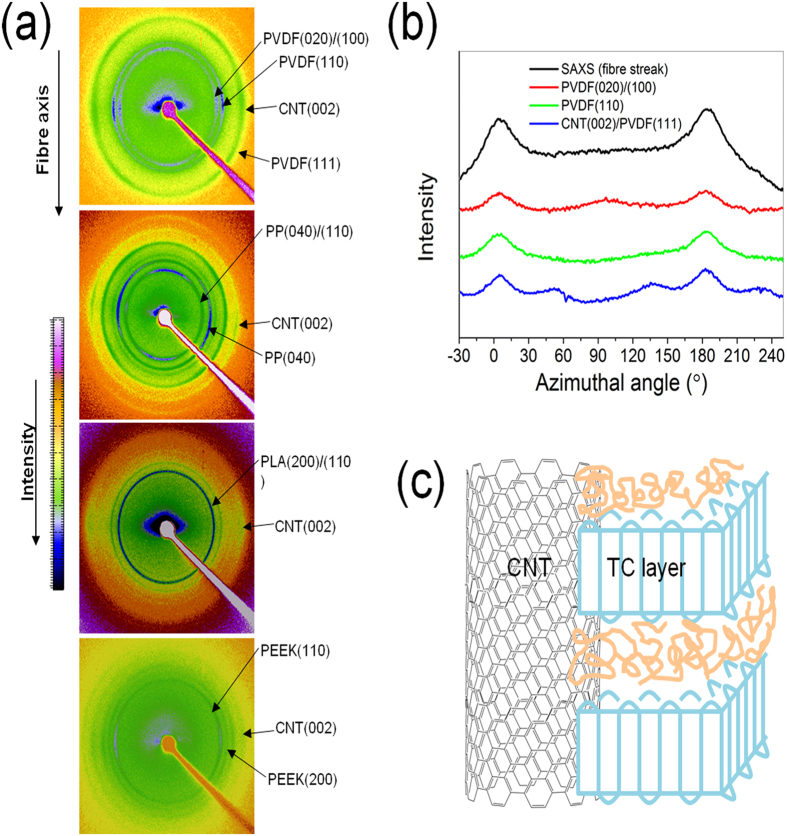
Orientation of polymer lamella with the chain axis predominantly parallel to the CNT axis. (**a**) WAXS pattern of four composites (PVDF-, PP-, PLA- and PEEK-CNT fibre) and (**b**) an example of the azimuthal profile from radial integration showing parallel orientation between the CNTs [(002) reflection and SAXS fibre streak] and the polymer (PVDF) principal axis. (**c**) Schematic illustrating the orientation between the CNT and TC lamella.

Wetting is seen as a requisite for heterogenous nucleation, but epitaxy and in general the specific preferential arrangement of the chains at the CNT fibre/polymer interface are expected to dictate the growth of the TC layer. Surprisingly, our WAXS measurements indicate that in all the composites in this work the lamella that make up of the TCL are oriented with the chain axis parallel to the fibre axis, that is, parallel to the CNTs. Figure 5 presents 2D WAXS patterns of the different composites, and azimuthal profiles from the PVDF/CNT fibre composite as an example (the azimuthal profiles for other polymers are included in Supplementary Fig. S7 online). The degree of alignment of CNTs and polymer lamellae extracted from the full width at half maximum of the azimuthal profiles is comparable (e.g. (002)CNT-20°, (110)PVDF-25°), as observed in somewhat similar CNT/PVA wet-spun composite fibres^33^. However, we note that our measurements correspond to unidirectional arrays containing ~3,800 individual fibre filaments with additional misalignment arising from their assembly. Accurate comparison of the orientation of the TC layer and its corresponding CNT fibre requires measurements on individual filaments using synchrotron radiation.

All the patterns show equatorial reflections of the (002) from the CNTs, corresponding to reflections from graphitic basal planes which are preferentially orientated parallel to the fibre axis and on which the polymer TC layer nucleates. PVDF exhibits the pseudo-orthorhombic α phase, with (110) and (020/100) reflections on the equator, corresponding to chain folding in the TC layer parallel to the CNTs. Because the CNT (002) overlaps with PVDF (111) we also include the azimuthal profile of the SAXS fibre streak, which has equivalent orientation as the basal planes^34^. In the PP/CNT composite the TC has the same preferential orientation, observed as the equatorial (110) and (040) reflections parallel to the CNT (002). The presence of daughter lamella grown on the parent PP TC layer produces additional (110) arcs on the meridian and contributes to equatorial (040), in agreement with previous reports^5^. PLA crystallises in the orthorhombic α phase with the main (200/110) reflection of higher intensity on the equator. It is consistent with the orientation of PP and PVDF lamella, but with a lower degree of orientation due to a smaller fraction of interfacial/bulk crystalline domains consistent with DSC data. PEEK, included in Fig. 5, has similar orientation of the orthorhombic form I phase with polymer chains oriented parallel to the fibre axis (see (110) and (200) reflections on the equator) and further shows that CNT-induced TC layer orientation is not very dependent on the specificity of the polymer. Experimental evidence to rule out the possibility of sample preparation externally inducing polymer orientation is presented in Supplementary Fig. S6 online.

To summarise the WAXS/SAXS data, it shows that CNT (002) reflections are parallel to polymer (*hk*0) and thus, that the orientation of polymer chains relative to the CNTs is equivalent for all of them. The reflections resulting arrangement of lamella and CNT observed for all the polymers is shown in Fig. 5c. The fact that it is the same for all the polymers studied here is very revealing. Polymers crystallisation on oriented films is typically explained in terms of lattice matching in one of the crystal directions^35^. Yet in the systems studied here such matching is unlikely in view of the wide range of unit cell parameters [a: 4.96 (PVDF)^36^– 10.7 (PLA)^37^, b: 5.9 (PEEK)^38^– 20.96 (PP)^39^, c: 4.62 (PVDF)^36^– 28.9 (PLA)^37^ Å]. Instead, the epitaxy observed in our CNT fibre/polymer composites is likely to be the result of polymer chains in the melt orienting parallel to the CNTs due to the high degree of CNT alignment and their diameter (predominantly 5 nm) being close to the radius of gyration of the polymer chain, forcing the polymer to adsorb parallel to the CNTs. This mechanism would be similar to that observed in nano shish-kebab structures prepared from solution crystallisation, termed soft epitaxy^11^, also obseved in PE crystallisation from solution in the presence of aligned CNT fibres^40^,^41^. It is also possible that shear forces induced by anisotropic capillary flow into the elongated fibre pores contribute to orienting polymer chains, however this alignment would have to be preserved during crystal melting and recrystallisation.

The reported observation of a wide range of polymers that nucleate on the surface of dispersed CNTs, either from solution (PE, P3HT, Nylon, PVA, PEG, PTFE) or from the melt (PP, PLA, PVDF)^13^, would further support the view that epitaxy in terms of crystallographic registry, lattice type of size matching is not a prerequisite for TC formation on CNT fibres, in agreement with some studies on surface nucleation of semicrystalline polymers^42^. With vast evidence that most polymer wet graphitic surfaces and that polymer chains tend to orient preferentially along the axis of CNTs, it would appear that heterogeneous nucleation at the surface of nanocarbons is predominant in these system and should be expected under appropriate kinetic conditions (e.g. dilute solution or slow cooling). In the case of fibres of aligned CNTs, when cooling from the melt, this orientation would be imparted onto a mesomorphic layer of polymer with properties between the crystal and the melt and which precedes the growth of lamella^43^, similar to the behavior observed in PE crystals growth from solution on pyrolytic graphite^44^ or on CNT fibres^40^,^41^.

## Conclusion

Polymer crystallisation is accelerated by the presence of macroscopic fibres of CNTs and results in the growth of a transcrystalline layer perpendicular to the fibre surface for a variety of semicrystalline polymers (PVDF, PP, PLA, PEEK) in spite of their differences in chemical make-up. The heterogeneous nucleation of these polymers is monitored (except PEEK) by POM, DSC and *in-situ* XRD measurements. In DSC, the TC is manifested as a clear crystallisation peak at higher temperatures for the composite samples compared to the bulk polymer. Extracting an induction time from isothermal crystallisation experiments we also compare the kinetics of interfacial (i.e. TC) and bulk (i.e. spherulitic) nucleation and confirm the former to proceed much faster.

2D WAXS measurements on the CNT fibre/polymer composite samples show the same preferential orientation of the polymer lamella with the chain axis parallel to the fibre and thus to the CNTs. This observation suggests that epitaxy in terms of lattice type or size matching is not critical for the nucleation of the TC layer. Instead, crystal growth at the CNT-polymer interface is associated with polymer chain alignment induced upon adsorption, in agreement with the soft-epitaxy model for solution crystallisation^11^ while also resembling the formation of a mesomorphic phase proposed in melt crystallisation^43^. In our composite systems, in addition to the small diameter of the CNTs inherently inducing such polymer alignment, additional orientation could be partially imparted by the directional flow of the polymer state in molten during infiltration into the elongated CNT fibre pores. These two possibilities will be analyzed in future studies.

## Methods

The CNT fibres were synthesized by the direct spinning method^45^. Briefly, the fibres are produced by continuous drawing of an aerogel of CNTs directly from the gas phase during CNT growth by chemical vapor deposition in a vertical reactor. Butanol, ferrocene and thiophene were used as carbon source, catalyst and promoter, respectively, in a concentration of 97.7:1.5:0.8 chosen so as to produce fibre made up of thin multiwall carbon nanotubes (MWNT) with average diameter of 5 nm^14^. PLA (PLA2002D), PP (HD120MO), and PVDF were purchased from NatureWorks, Borealis, and Sigma-Aldrich with weight-average molecular weights of 210,000 g/mol, 365,000 g/mol, and 180,000 g/mol, respectively. Poly(ether ether ketone) (PEEK) was purchased from Ketaspire KT-820 NT, Solvay Plastics.

Composites of PVDF-, PP-, PEEK- and PLA-CNT fibres were hot-pressed on a hot-plate consolidate machine (LabPro 400, Fontijne Presses) equipped with a water cooling pump, as follows. First, a macroscopic CNT fibre film consisting of ~3,800 individual fibre filaments was produced using the direct spinning route described above and winding parallel filaments onto a rotating spindle. This unidirectional fibre film was then sandwiched between two sheets of similar size of thin polymer film, which were positioned together into a metal frame to control thickness of the final sample. Next, the sandwiched sample was heated at 10˚C/min up to the polymer melting temperature, at which point a compressive force of 5 kN was applied and held for around 5 minutes. The sample was subsequently cooled down (at 10˚C/min) to room temperature to release the composite. The resulting polymer-CNT fibre composites have dimensions of about 50 mm (length), 50 mm (width) and tens of micrometers (thickness). These samples were cut into different shapes depending on the requirements of the various characterisation techniques used. For comparison, pure polymers (PVDF, PP, PLA and PEEK) were prepared under the same processing conditions as the equivalent CNT fibre composites. Mass fractions of CNT fibre in three composites were determined by thermogravimetric analysis and sample weighing, giving typical values of 10 to 15 wt% for hot-pressed samples. For all the polymers types, control samples were produced by melting and recrystallisation after initial preparation in order to eliminate potential pre-orientation induced during hot-pressing. Further control samples of CNT film, onto which ground polymer was manually deposited, then melted in an oven above the crystallisation temperature and cooled down slowly without applying any external pressure to promote infiltration, were also produced.

Constant cooling DSC measurements of plain polymers and composites were carried out by slow cooling from the melt at 10˚C/min and performed with a Q200 TA Instruments DSC equipped with a cooling pump. Isothermal studies: Samples were cooled down from the melt at 20 °C/min to an isothermal temperature (in supercooling state), followed by a kinetic recording of exothermal heat flows (see Supplementary Fig. S1 online). At least four isothermal temperatures for each polymer system were used in order to calculate nucleation rates. For POM observation, samples of a continuous CNT fibre embedded between thin polymer films and supported on a glass slide, were subjected to an isothermal condition. Their nucleation/crystallisation processes was monitored with an optical microscope (Carl Zeiss Jena with a CCD-IRIS camera, Sony) equipped with a hot stage (THMS600, controlled by Linkam TMS92). The morphology of composite samples was observed with a FIB-FEGSEM microscope (Helios NanoLab 600i FEI) at an accelerating voltage of 5 kV. A thin layer of gold was sputter-coated on the surface of these specimens.

Conventional wide-angle X-ray diffraction photographs were recorded on a flat-plate camera attached to a Phillips 2 kW tube X-ray generator (nickel-filtered Cu Kα radiation, λ = 1.54 Å) with sample to detector distance of 85 mm. 2D WAXS/SAXS patterns were also collected at NCD beamline11, λ = 1.24 Å, (ALBA Synchrotron Light Facility, Spain) every 2 °C during *in-situ* melting/cooling cycles at a rate of 10 °C/min using a Linkam controller. The data shown are after sample-detector distance calibration. Twenty-degree sections of the 2D patterns were azimuthally integrated parallel and perpendicular to the fibre axis to obtain radial profiles for the bulk and TC.

## Additional Information

**How to cite this article**: Yue, H. *et al*. Macroscopic CNT fibres inducing non-epitaxial nucleation and orientation of semicrystalline polymers. *Sci. Rep*. **5**, 16729; doi: 10.1038/srep16729 (2015).

## Supplementary Material

Supplementary Information

## Figures and Tables

**Table 1:** Surface energy difference (γ_*SL*_ - γ_*S*_) upon wetting of the CNT fibre by different polymers.

	**Graphite**	20 nm MWNT
	(γ_*S*_* = *γ_*S*_^*d*^ = 79 mJ/m^2^)	(γ_*S*_^*d*^ = 17.6 mJ/m^2^, γ_*S*_^*p*^ = 10.2 mJ/m^2^)
PLA	−23.9	−56.8
PVDF	−20.2	−47.7
PP	−34.0	−68
