# Supplementary material for: Macroscopic CNT fibres inducing non-epitaxial nucleation and orientation of semicrystalline polymers
Source: Sci Rep. 2015 Nov 18;5:16729. doi: 10.1038/srep16729 (PMC4649745; doi:10.1038/srep16729)
Supplement: Supplementary Information [file srep16729-s1.pdf]

Supporting Information

Macroscopic CNT fibres inducing non-epitaxial nucleation and orientation of semicrystalline polymers

Hangbo Yue[1], Alfonso Monreal-Bernal[1], Juan P. Fernández-Blázquez[1], Javier Llorca[1,2], and Juan J. Vilatela[1,*]

[1] IMDEA Materials Institute, Eric Kandel 2, Getafe, Madrid 28906, Spain
[2] Department of Materials Science, Polytechnic University of Madrid, 28040 Madrid, Spain
* juanjose.vilatela@imdea.org

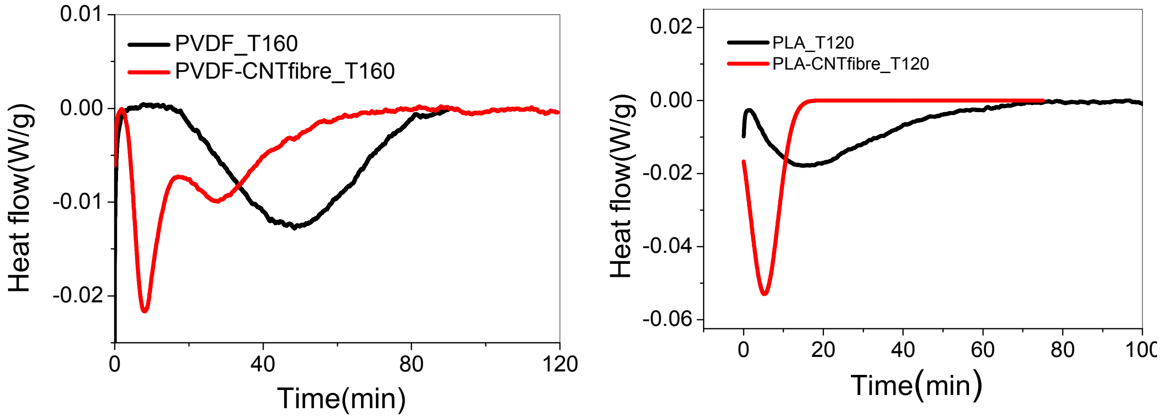

Supplementary Figure S1. Examples of isothermal heat flows for semi-crystalline polymers (PVDF and PLA) and the CNT fibre-polymer composites, at isothermal temperature of 160 ºC and 120 ºC, respectively.

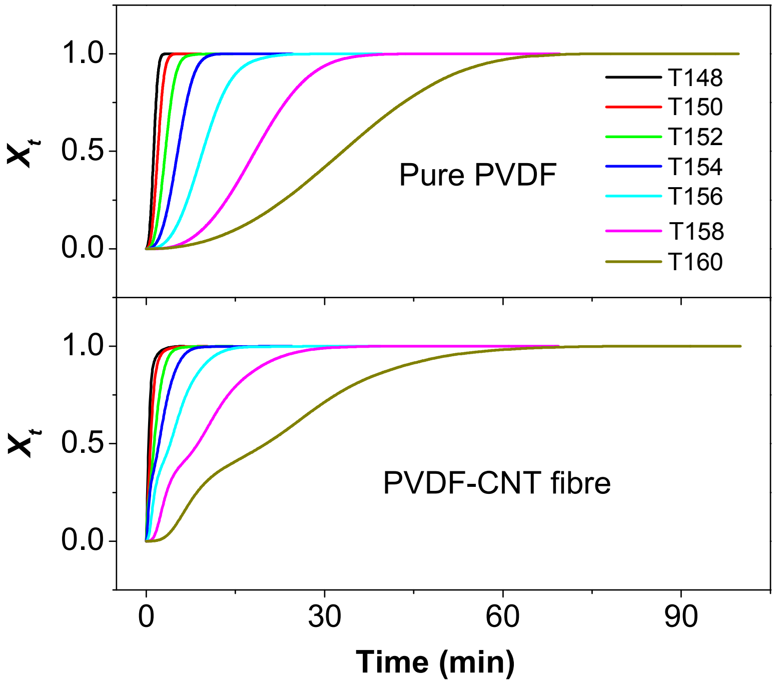

Supplementary Figure S2. Plots of relative crystallinity (X_t) as a function of time during isothermal crystallisation of polymer (PVDF) and the CNT fibre based composite at various temperatures.

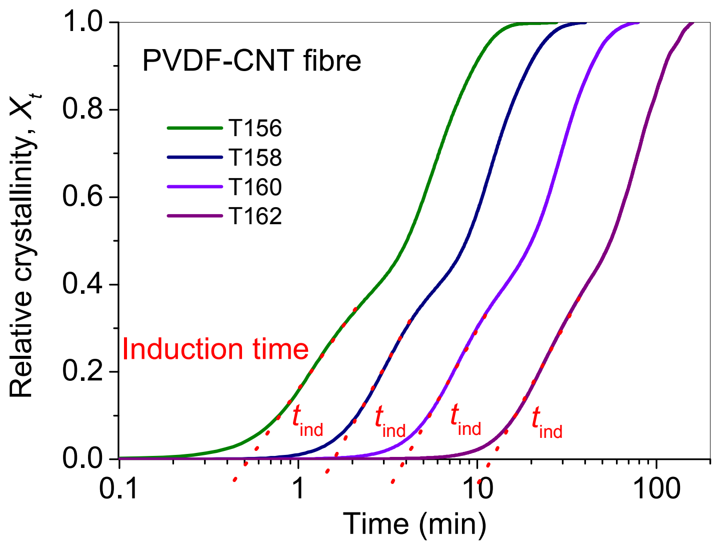

Supplementary Figure S3. An example showing the acquisition of induction time, t_{ind}, by extrapolating X_t=0 from plots of relative crystallinity X_t against time t in logarithm scale.

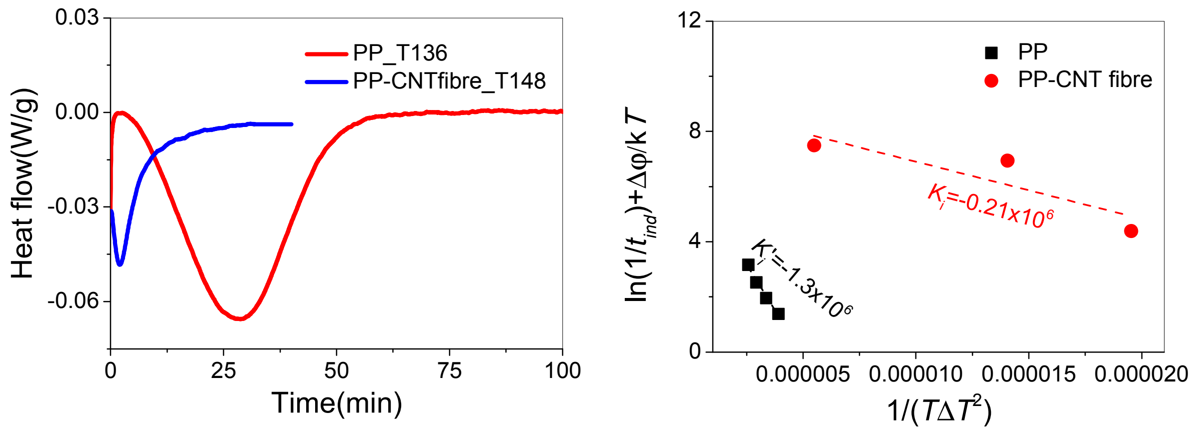

Supplementary Figure S4. DSC isothermal heat flows (left) and plots of ln*(1/ t_{ind})+ Δφ/kT vs 1/(T*Δ*T*^{2}*)* (right) for PP and the PP-CNT fibre composites.

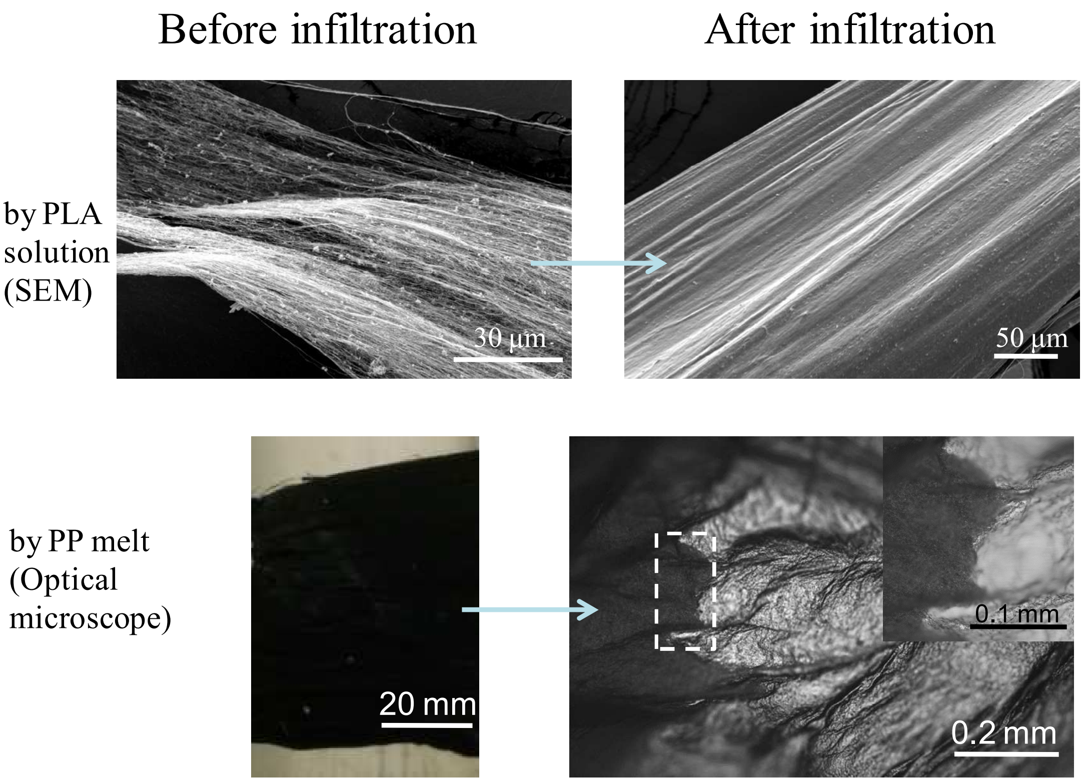

Supplementary Figure S5. Images of CNT fibres and that wet by polymer solution and melt. PLA solution was prepared by dissolving PLA in dichloromethane, and solid PP was heated into the molten state via heating treatment.

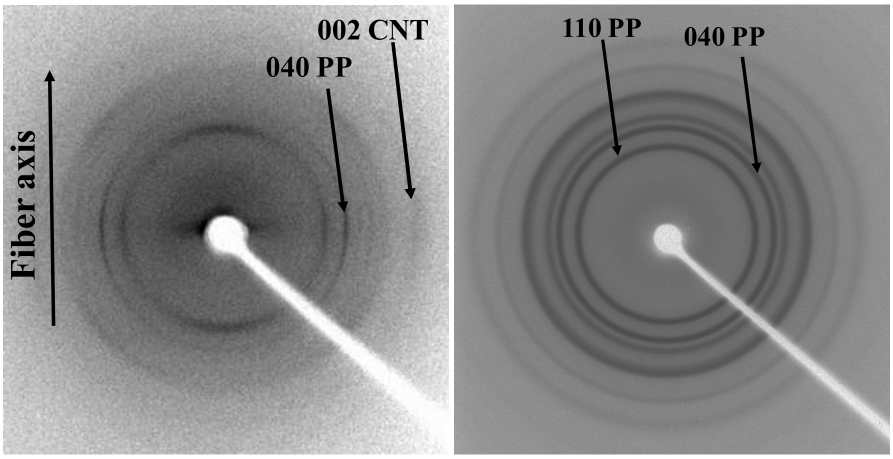

Supplementary Figure S6. WAXS pattern of PP/CNT fibre sample (left) prepared without any external pressure applied during melting, and WAXS patterns of pure polymers (right) showing no preferential orientation of the crystalline domains.

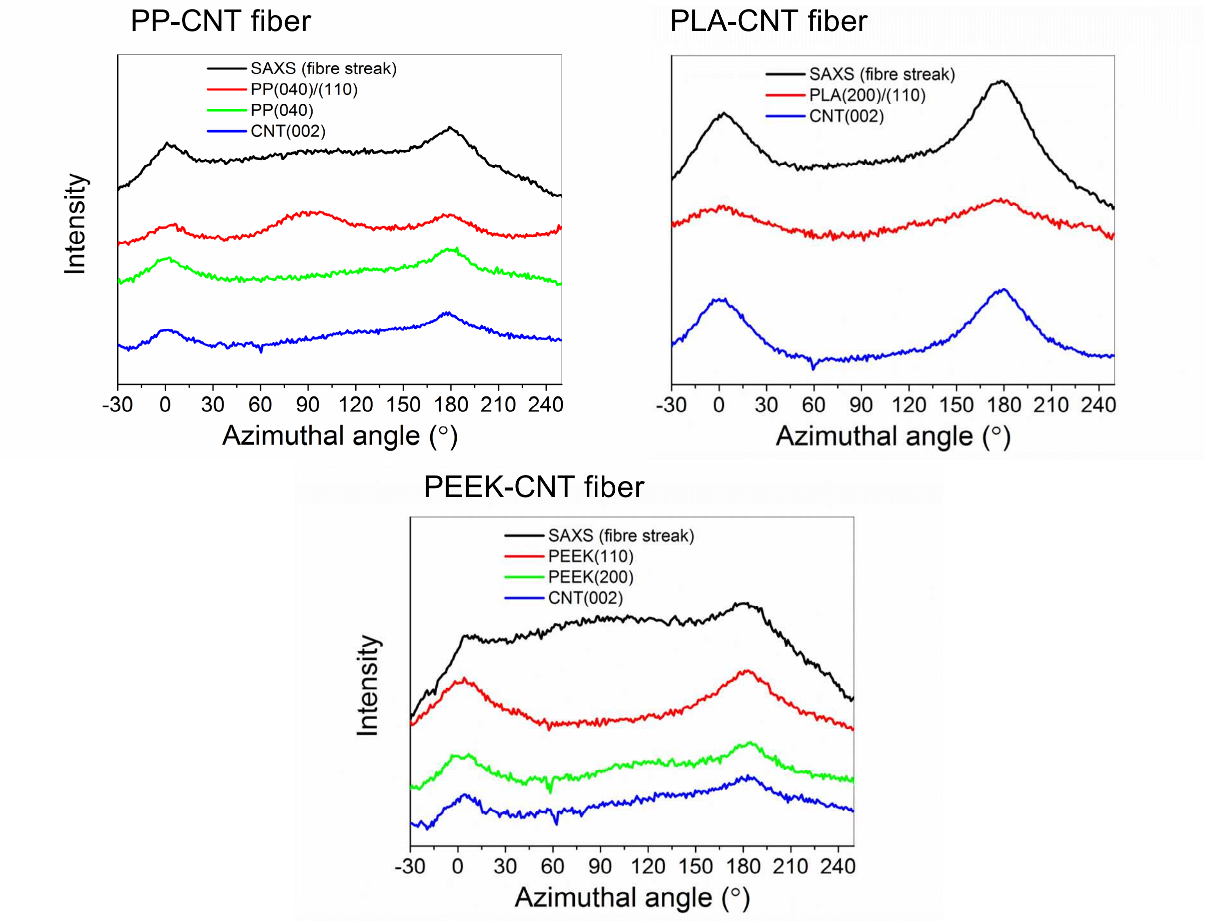

Supplementary Figure S7. Azimuthal profiles of the relevant reflections, from the semi-crystalline polymers, the SAXS fibre streak, and the CNT (002).

Supplementary Table S1. Structural formula, polarity, unit cell parameters, and total γ (dispersion γ^d plus polar γ^p) surface free energy of polymers (PLA, PP, PVDF, PEEK).

	PLA	PP	PVDF	PEEK	References
structural formula	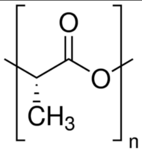	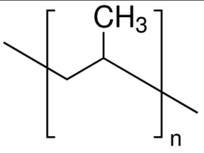	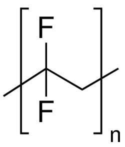	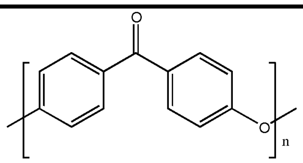	
polarity	polar	non polar	polar	non polar	
unit cell	orthorhombic	monoclinic	monoclinic	orthorhombic	1, 2, 3, 4
a (Å)	10.68	6.65	4.96	7.8	1, 2, 3, 4
b (Å)	6.17	20.96	9.64	5.92	1, 2, 3, 4
c (Å)	28.86	6.5	4.62	10.05	1, 2, 3, 4
γ	40.7	32.1	30.3	42.1	5, 6
γ^d	32.5	31.6	23.3	36.2	5, 6
γ^p	8.2	0.5	7	5.9	5, 6

Supplementary Table S2. Hansen solubility parameters and calculated Hansen space *R*.

	δ^{d}	δ^{p}	δ^{h}	R	Reference
CNT	18.6	10.1	7.5	--	7
PLA	18.5	9.7	6	1.6	8
PP	18	3	3	8.5	9
PVDF	19.4	15.9	11.3	7.1	10

DSC isothermal measurements

To calculate the nucleation and growth rate, DSC isothermal analysis of the samples was made using TA Instruments DSC Q200 under nitrogen atmosphere. The thermal history was programmed as follows: (i) the sample (~5 mg) was heated at a heating rate of 10 ºC/min to a point (~200 ºC) above polymer melting temperature; (b) kept at this temperature for 2 min; (c) cooled the sample down from the melt to an isothermal temperature (polymer in supercooling state) at a cooling rate of 20 ºC/min. (d) since then the heat flows were immediately recorded as a function of time, as shown in Fig. S1.

Method of induction time acquisition

The onset of TC growth in Fig. 4 is taken as the induction time for nucleation t_{ind}, which through its temperature dependence can be used to calculate nucleation rate constants. Specifically, from the typical growth curve (Fig. S3), extrapolating to X_t=0 we obtain an induction time, t_{ind}, which we assume to correspond to the induction time for nucleation [11]. This is equivalent to assuming that the onset for nucleation and the onset for growth detected by DSC are the same. This is a reasonable assumption for the purposes of comparing nucleation rates for the TC and bulk because a) the interest is in the temperature dependence of the induction time rather than in the absolute values of t_{ind} and b) the rates of TC and spherulitic growth are nearly the same (as shown in the main text).

Heterogeneous nucleation rate for PP samples

Due to very fast nucleation, crystallisation of PP-CNT is accelerated to the point that TC growth rates cannot be accurately extracted from the DSC data (Fig. S4). Nevertheless, the slope of $\ln(1/\ t_{ind}) + \Delta\varphi/kT$ *vs* $1/(T\Delta T^2)$ representing the nucleation kinetic constants K_i still indicates an accelerated nucleation rate in the fibre composite compared to neat PP.

Evidence of polymer wetting CNT fibres

Three polymers (PLA, PP and PVDF) in this work, when in molten state or solution, readily infiltrate the porous fibre structure and wick, by replacing air and the surface of CNT exposed to the surrounding. As a result, a coating layer of polymer on the surface of CNT fibre was formed, as shown in Fig. S5.

Analysis of Hassen affinity parameter

Polymer/CNT fibre affinity is calculated as the distance in Hansen space according to Bergin et al: [7]

$$R = \sqrt{\left(\delta_{CNT}^{d} - \delta_{polym}^{d}\right)^2 + \left(\delta_{CNT}^{p} - \delta_{polym}^{p}\right)^2 + \left(\delta_{CNT}^{h} - \delta_{polym}^{h}\right)^2} \qquad \text{(S1)}$$

where δ^{i} are the d=dispersive, p=polar and h=hydrogen components of the Hansen solubility parameter of the CNT and polymer. Solvents that interact strongly with CNTs give values of R in the range $1<R<10$. Table S2 lists Hansen solubility parameters of CNT and the three polymers (PLA, PP and PVDF) and values of the polymer-CNT distance in Hansen space (R_{PLA}=1.6, R_{PP}=8.5, R_{PVDF}=7.1 MPa$^{1/2}$) in a “wetting” range ($1<R<10$).[7]

Orientation of polymer chains

For all the polymers types, the composite samples were recrystallized to remove thermal history and confirm the orientation relation in Fig. 5. Furthermore, we produced a CNT film onto which ground PP was manually deposited, then melted in an oven above the crystallisation temperature and cooled down slowly without applying any external pressure to promote infiltration. The sample presented in Fig. S6 shows the same orientation as that in Fig. 5. Finally, control samples of pure polymer were also analyzed. Their WAXS patterns correspond to rings and confirm that the samples are isotropic.

Supplementary References

1. Wasanasuk K., *et al.* Crystal structure analysis of poly(l-lactic acid) α form on the basis of the 2-dimensional wide-angle synchrotron X-ray and neutron diffraction measurements. *Macromolecules* **44**, 6441-6452 (2011).

2. Natta G., Corradini P. Structure and properties of isotactic polypropylene. *Il Nuovo Cimento Series 10* **15**, 40-51 (1960).

3. Hasegawa R., Takahashi Y., Chatani Y., Tadokoro H. Crystal structures of three crystalline forms of poly(vinylidene fluoride). *Polym. J.* **3**, 600-610 (1972).

4. Liu T., Wang S., Mo Z., Zhang H. Crystal structure and drawing-induced polymorphism in poly(aryl ether ether ketone). Iv. *J. Appl. Polym. Sci.* **73**, 237-243 (1999).

5. Khoshkava V., Kamal M.R. Effect of surface energy on dispersion and mechanical properties of polymer/nanocrystalline cellulose nanocomposites. *Biomacromolecules* **14**, 3155-3163 (2013).

6. Wu S. Calculation of interfacial tension in polymer systems. *Journal of Polymer Science Part C: Polymer Symposia* **34**, 19-30 (1971).

7. Bergin S.D., Sun Z.Y., Rickard D., Streich P.V., Hamilton J.P., Coleman J.N. Multicomponent solubility parameters for single-walled carbon nanotube-solvent mixtures. *ACS Nano* **3**, 2340-2350 (2009).

8. Agrawal A., Saran A.D., Rath S.S., Khanna A. Constrained nonlinear optimization for solubility parameters of poly(lactic acid) and poly(glycolic acid)—validation and comparison. *Polymer* **45**, 8603-8612 (2004).

9. Hansen C.M. *Hansen solubility parameters: A user's handbook.* CRC press (2012).

10. Bottino A., Capannelli G., Munari S., Turturro A. Solubility parameters of poly(vinylidene fluoride). *J. Polym. Sci., Part B: Polym. Phys.* **26**, 785-794 (1988).

11. Ishida H., Bussi P. Surface induced crystallization in ultrahigh-modulus polyethylene fiber-reinforced polyethylene composites. *Macromolecules* **24**, 3569-3577 (1991).
